# A one-pot catalyst-free synthesis of functionalized pyrrolo[1,2-*a*]quinoxaline derivatives from benzene-1,2-diamine, acetylenedicarboxylates and ethyl bromopyruvate

**DOI:** 10.3762/bjoc.9.55

**Published:** 2013-03-11

**Authors:** Mohammad Piltan, Loghman Moradi, Golaleh Abasi, Seyed Amir Zarei

**Affiliations:** 1 Department of Chemistry, Sanandaj Branch, Islamic Azad University, Sanandaj, Iran; 2 Department of Chemistry, University of Kurdistan, Sanandaj, Iran

**Keywords:** acetylenedicarboxylates, benzene-1,2-diamine, ethyl bromopyruvate, pyrrolo[1,2-*a*]pyrazine, pyrrolo[1,2-*a*]quinoxaline

## Abstract

The catalyst-free multicomponent reaction of 1,2-diaminobenzene, dialkyl acetylenedicarboxylates, and ethyl bromopyruvate forms pyrrolo[1,2-*a*]quinoxaline derivatives in good yields. Ethylenediamine also reacts under similar conditions to produce new pyrrolo[1,2-*a*]pyrazine derivatives.

## Introduction

Among the various classes of heterocyclic compounds, quinoxalines, a class of N-containing heterocycles, form an important component of many pharmacologically active compounds [1–4]. For example, the quinoxaline ring is a constituent of various bioactive compounds that possess antibiotic, anti-inflammatory, antimicrobial [5], antidiabetic [6], and antiviral activity against retroviruses including HIV [7]. In addition, quinoxaline derivatives are also associated with a wide spectrum of biological effects including anticancer [8], antifungal, and antidepressant activities [9–10]. Two commercially available antibiotic families of quinoxalines, echinomycin [11–12] and triostins [13], are well known. Hence, the synthesis of quinoxaline derivatives is currently of significant interest in organic synthesis. As an important quinoxaline derivative, the pyrrolo[1,2-*a*]quinoxaline moiety, in particular, is a tricyclic compound having biological activity [14–19]. The syntheses of pyrrolo[1,2-*a*]quinoxaline derivatives have hitherto been reported by only a few researchers, and they normally required additional additives and long reaction times and displayed limited reaction tolerance and low reaction selectivity [20–21]. Cheeseman and Tuck reported the first pyrrolo[1,2-*a*]quinoxaline in 1965 [22]. Recently, Patil et al. developed the PtBr_2_ and Au(I)-catalyzed hydroamination–hydroarylation cascade reactions of 2-(1*H*-pyrrol-1-yl)anilines with alkynes to form 4,5-dihydropyrrolo[1,2-*a*]quinoxalines [23–24]. Kobayashi and co-workers have described the Lewis acid catalyzed cyclization of 1-(2-isocyanophenyl)pyrroles to give pyrrolo[1,2-*a*]quinoxalines in good yields; however, the isocyanide substrates required multistep synthesis [25–26]. Ma and Yuan have reported the synthesis of pyrrolo[1,2-*a*]quinoxalin-4(5*H*)-ones by CuI/L-proline-catalyzed coupling of *N*-trifluoroacetyl-2-haloanilines with methyl pyrrole-2-carboxylates [27]. The development of more efficient methods for the preparation of these compounds is still an active research area. The syntheses of pyrrolo[1,2- *a*]isoquinolines have previously been reported by us [28]. In this paper, we describe a simple synthesis of functionalized pyrrolo[1,2-*a*]quinoxaline and pyrrolo[1,2-*a*]pyrazine derivatives in the absence of catalysts.

## Results and Discussion

The reaction of 1,2-diaminobenzenes **1** with dialkyl acetylenedicarboxylates **2a–c** in the presence of ethyl bromopyruvate (**3**) was performed in acetonitrile under reflux over 12 hours. The ^1^H and ^13^C NMR spectra of the crude products clearly indicated the formation of polysubstituted pyrrolo[1,2-*a*]quinoxaline derivatives **4a–h** in 88–93% yields (Table 1). The structures of products **4a–h** were established from ^1^H NMR, ^13^C NMR and IR spectra and MS. For example, the ^1^H NMR spectrum of **4a** exhibited a singlet at 3.78 ppm for the methyl ester group, and one quartet at 4.20 ppm and one triplet at 1.21 ppm for the ethoxy group, along with multiplets (7.08–8.70 ppm) for the aromatic region, and a broad singlet at 11.51 ppm due to the *NH* group. The proton-decoupled ^13^C NMR spectrum of **4a** showed 16 distinct resonances in agreement with the proposed structure. The IR spectrum of **4a** displayed characteristic carbonyl bands (1730, 1726 and 1695 cm^−1^). The ^1^H NMR and ^13^C NMR spectra of products **4b–h** were similar to those of **4a**, except for the ester moieties, which exhibited characteristic resonances in the appropriate regions of the spectrum. Substitution on the benzene-1,2-diamine was also found to be important. A methyl group provided good yields (Table 1, entries d, e and h), while a nitro group did not participate in the reaction (Table 1, entries f and g). It seems that the high electron-withdrawing effect of the nitro group deactivates the amine.

**Table 1:** Syntheses of pyrrolo[1,2-*a*]quinoxalines **4**.

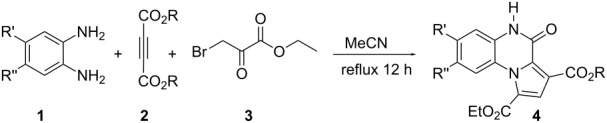				

Entry	Activated acetylene	Diamine	Product	Yield (%) ^a^

a	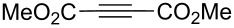 **2a**	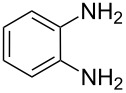 **1a**	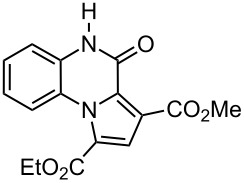 **4a**	91
b	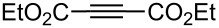 **2b**	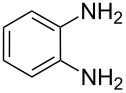 **1a**	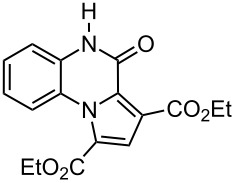 **4b**	90
c	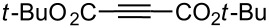 **2c**	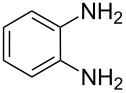 **1a**	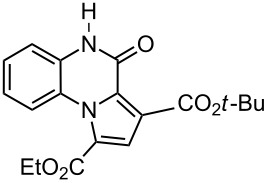 **4c**	88
d	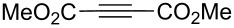 **2a**	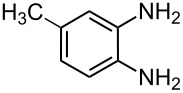 **1b**	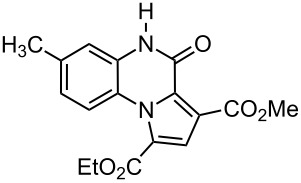 **4d**	92
e	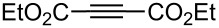 **2b**	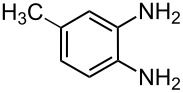 **1b**	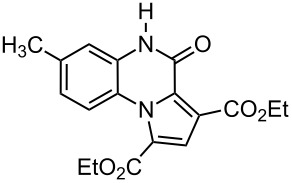 **4e**	91
f	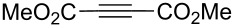 **2a**	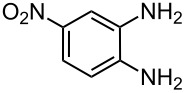 **1c**	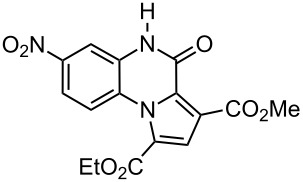 **4f**	0^b^
g	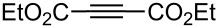 **2b**	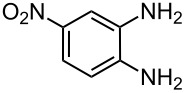 **1c**	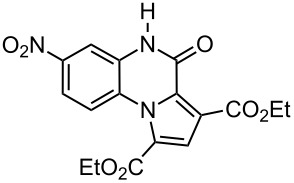 **4g**	0^b^
h	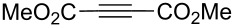 **2a**	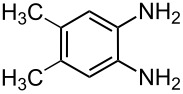 **1d**	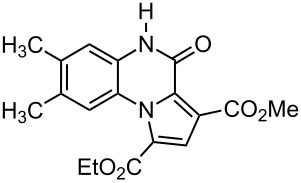 **4h**	93

^a^Isolated yield. ^b^The reaction was carried out for 24 h.

**Scheme 1:**
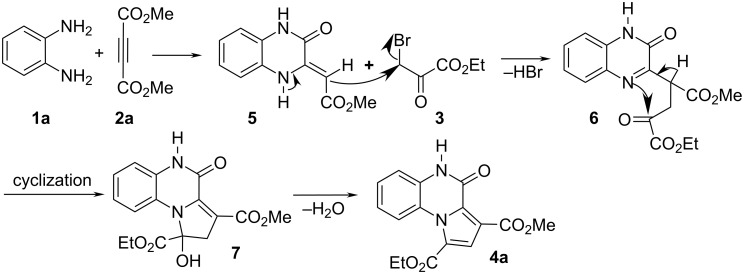
Proposed mechanism for the formation of compound **4a**.

Although the mechanistic details of the reaction are not known, a plausible mechanism maybe put forward to explain the product formation (Scheme 1). On the basis of the well-established chemistry of amines and DMAD [29–30], the reaction between 1,2-diaminobenzene and dimethyl acetylenedicarboxylate (**2a**) affords dihydroquinoxaline **5**. Compound **5** possesses enamine character and, thus, can readily react with ethyl bromopyruvate (**3**) to generate the intermediate **6**. This intermediate undergoes a series of cyclization and elimination reactions to generate the product **4a**.

**Scheme 2:**
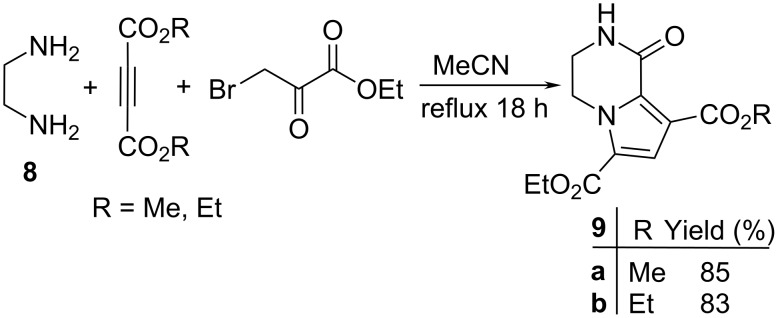
Synthesis of pyrrolo[1,2-*a*]pyrazine derivatives **9a**,**b**.

We then investigated the reaction between ethylenediamine (**8**) and activated acetylenic compounds in the presence of ethyl bromopyruvate under similar conditions to form pyrrolo[1,2-*a*]pyrazine derivatives (Scheme 2). Compounds **9a,b** were fully characterized according to their elemental analyses and their IR, ^1^H NMR and ^13^C NMR spectra and MS.

## Conclusion

In summary, the reaction between benzene-1,2-diamine and dialkyl acetylenedicarboxylates in the presence of ethyl bromopyruvate provides a simple, catalyst-free one-pot entry to the synthesis of pyrrolo[1,2-*a*]quinoxaline derivatives having potential synthetic and pharmacological interest. The simplicity of the present procedure makes it an interesting alternative to other approaches.

## Experimental

### General

The reagents and solvents used in this work were obtained from Aldrich and Fluka and were used without further purification. Mp: Electrothermal-9100 apparatus. IR spectra (KBr): Shimadzu IR-460 spectrometer; in reciprocal centimeters (cm^−1^). ^1^H and ^13^C NMR spectra: Bruker DRX-250.1 Avance instrument; in DMSO-*d*_6_ at 250.1 and 62.9 MHz, resp.; δ in parts per million (ppm), *J* in hertz (Hz). MS: Finnigan-MAT-8430 mass spectrometer at 70 eV; in *m/z* (rel. %). Elemental analyses (C, H, N): HeraeusCHN-O-Rapid analyzer.

#### General procedure for the synthesis of functionalized pyrrolo[1,2-*a*]quinoxalines **4**

To a stirred solution of **1** (2 mmol) and **2** (2 mmol) in MeCN (5 mL) was added **3** (2 mmol) at rt. The mixture was heated under reflux for 12 h. The resulting precipitate was filtered off and recrystalized from MeOH to give **4**.

**1-Ethyl 3-methyl 4-oxo-4,5-dihydropyrrolo[1,2-*a*]quinoxaline-1,3-dicarboxylate (4a):** Grey solid, mp 247–249 °C (dec), 0.57 g, yield 91%; IR (KBr) (v_max_/cm^−1^): 3290 (NH), 1730 (C=O), 1726 (C=O), 1695 (C=O), 1282 (C–O); ^1^H NMR (250.1 MHz, DMSO-*d*_6_) δ 1.21 (t, ^3^*J* = 7.1 Hz, 3H, CH_3_), 3.78 (s, 3H, OCH_3_), 4.20 (q, ^3^*J* = 7.1 Hz, 2H, OCH_2_), 7.08–7.17 (m, 3H, CH), 8.14 (d, ^3^*J* = 8.2 Hz, 1H, CH), 8.70 (s, 1H, CH), 11.51 (s, 1H, NH) ppm; ^13^C NMR (62.9 MHz, DMSO-*d*_6_) δ 14.5 (CH_3_), 53.1 (OCH_3_), 61.2 (OCH_2_), 115.2 (CH), 118.1 (C), 119.7 (CH), 120.8 (C), 121.0 (C), 122.0 (C), 122.1 (C), 123.5 (CH), 127.5 (CH), 129.7 (CH), 155.1 (C=O), 162.8 (C=O), 165.1 (C=O) ppm; EIMS *m*/*z*: 314 (M^+^, 100), 299 (7), 285 (9), 283 (14), 269 (36); anal. calcd for C_16_H_14_N_2_O_5_ (314.29): C, 61.14; H, 4.49; N, 8.91; found: C, 61.28; H, 4.37; N, 8.97.

**Diethyl 4-oxo-4,5-dihydropyrrolo[1,2-*a*]quinoxaline-1,3-dicarboxylate (4b):** Grey solid, mp 245–247 °C (dec), 0.59 g, yield 90%; IR (KBr) (v_max_/cm^−1^): 3287 (NH), 1726 (C=O), 1724 (C=O), 1688 (C=O), 1286 (C–O); ^1^H NMR (250.1 MHz, DMSO-*d*_6_) δ 1.11 (t, ^3^*J* = 7.2 Hz, 3H, CH_3_), 1.36 (t, ^3^*J* = 7.2 Hz, 3H, CH_3_), 3.84 (q, ^3^*J* =7.2 Hz, 2H, OCH_2_), 4.29 (q, ^3^*J* = 7.2 Hz, 2H, OCH_2_), 7.12–7.36 (m, 3H, 3CH), 8.06 (d, ^3^*J* = 8.2 Hz, 1H, CH), 8.56 (s, 1H, CH), 11.51 (s, 1H, NH) ppm; ^13^C NMR (62.9 MHz, DMSO-*d*_6_) δ 14.5 (CH_3_), 14.6 (CH_3_), 61.2 (OCH_2_), 62.2 (OCH_2_), 115.1 (CH), 118.0 (C), 119.4 (CH), 120.7 (C), 121.0 (C), 122.0 (C), 122.1 (C), 123.6 (CH), 127.6 (CH), 129.6 (CH), 155.2 (C=O), 163.0 (C=O), 165.4 (C=O) ppm; EIMS *m*/*z*: 328 (M^+^, 100), 299 (12), 283 (95), 256 (72); anal. calcd for C_17_H_16_N_2_O_5_ (328.32): C, 62.19; H, 4.91; N, 8.53; found: C, 62.18; H, 4.83; N, 8.59.

**3-*tert*-Butyl 1-ethyl 4-oxo-4,5-dihydropyrrolo[1,2-*a*]quinoxaline-1,3-dicarboxylate (4c):** Grey solid, mp 233–235 °C (dec), 0.54 g, yield 88%; IR (KBr) (v_max_/cm^−1^): 3310 (NH), 1735 (C=O), 1728 (C=O), 1700 (C=O), 1281 (C–O); ^1^H NMR (250.1 MHz, DMSO-*d*_6_) δ 1.25 (s, 9H, C*Me*_3_), 1.34 (t, ^3^*J* = 7.2 Hz, 3H, CH_3_), 4.22 (q, ^3^*J* = 7.2, 2H, OCH_2_), 7.01–7.17 (m, 3H, 3CH), 8.08 (d, ^3^*J* = 8.1 Hz, 1H, CH), 8.58 (s, 1H, CH), 11.56 (s, 1H, NH) ppm; ^13^C NMR (62.9 MHz, DMSO-*d*_6_) δ 14.5 (CH_3_), 27.6 (C*Me*_3_), 61.6 (OCH_2_), 81.6 (*C*Me_3_), 114.9 (CH), 118.6 (C), 120.1 (CH), 120.8 (C), 121.5 (C), 122.3 (C), 122.9 (C), 123.2 (CH), 127.4 (CH), 129.1 (CH), 154.2 (C=O), 163.1 (C=O), 166.0 (C=O) ppm; EIMS *m*/*z*: 356 (M^+^, 100), 311 (62), 283 (54), 256 (48); anal. calcd for C_19_H_20_N_2_O_5_ (371.41): C, 64.04; H, 5.66; N, 7.86; found: C, 63.92; H, 5.57; N, 7.93.

**1-Ethyl 3-methyl 7-methyl-4-oxo-4,5-dihydropyrrolo[1,2-*a*]quinoxaline-1,3-dicarboxylate (4d):** Grey solid, mp 228–230 °C (dec), 0.60 g, yield 92%; IR (KBr) (v_max_/cm^−1^): 3295 (NH), 1728 (C=O), 1668 (C=O), 1618 (C=O), 1280 (C–O); ^1^H NMR (250.1 MHz, DMSO-*d*_6_) δ 1.23 (t, ^3^*J* = 7.1 Hz, 3H, CH_3_), 2.31 (s, 3H, CH_3_), 3.78 (s, 3H, OCH_3_), 4.22 (q, ^3^*J* = 7.1 Hz, 2H, OCH_2_), 7.02–7.12 (m, 2H, CH), 8.15 (d, 1H, ^3^*J* = 8.2 Hz, CH), 8.72 (s, 1H, CH), 11.51 (s, 1H, NH) ppm; ^13^C NMR (62.9 MHz, DMSO-*d*_6_) δ 14.1 (CH_3_), 21.2 (CH_3_), 52.7 (OCH_3_), 61.3 (OCH_2_), 115.4 (CH), 117.3 (C), 119.5 (CH), 120.1 (C), 121.3 (C), 122.5 (C), 122.9 (C), 123.7 (C), 127.1 (CH), 130.2 (CH), 154.6 (C=O), 162.5 (C=O), 164.8 (C=O) ppm; EIMS *m*/*z*: 328 (M^+^, 100), 313 (9), 297 (85), 256 (72); anal. calcd for C_17_H_16_N_2_O_5_ (328.32): C, 62.19; H, 4.91; N, 8.53; found: C, 62.11; H, 4.87; N, 8.64.

**Diethyl 7-methyl-4-oxo-4,5-dihydropyrrolo[1,2-*a*]quinoxaline-1,3-dicarboxylate (4e):** Grey solid, mp 249–251 °C (dec), 0.62 g, yield 91%. IR (KBr) (v_max_/cm^−1^): 3296 (NH), 1726, 1724, 1688, (C=O), 1286 (C–O); ^1^H NMR (250.1 MHz, DMSO-*d*_6_) δ 1.17 (t, ^3^*J* = 7.2 Hz, 3H, CH_3_), 1.36 (t, ^3^*J* = 7.2 Hz, 3H, CH_3_), 2.31 (s, 3H, CH_3_), 3.91 (q, ^3^*J* = 7.2 Hz, 2H, OCH_2_), 4.31 (q, ^3^*J* = 7.2 Hz, 2H, OCH_2_), 7.06–7.13 (m, 2H, 2CH), 8.11 (d, ^3^*J* = 8.2 Hz, 1H, CH), 8.61 (s, 1H, CH), 11.56 (s, 1H, NH) ppm; ^13^C NMR (62.9 MHz, DMSO-*d*_6_) δ 14.1 (CH_3_), 14.3 (CH_3_), 21.6 (CH_3_), 61.0 (OCH_2_), 61.7 (OCH_2_), 115.3 (CH), 118.4 (C), 119.1 (CH), 120.5 (C), 121.3 (C), 122.4 (C), 122.6 (C), 123.3 (C), 127.9 (CH), 129.2 (CH), 155.4 (C=O), 163.2 (C=O), 165.1 (C=O) ppm; EIMS *m*/*z*: 342 (M^+^, 100), 313 (14), 297 (91), 270 (68); anal. calcd for C_18_H_18_N_2_O_5_ (342.35): C, 63.15; H, 5.30; N, 8.18; found: C, 63.23; H, 5.36; N, 8.12.

**1-Ethyl 3-methyl 7,8-dimethyl-4-oxo-4,5-dihydropyrrolo[1,2-*a*]quinoxaline-1,3-dicarboxylate (4h):** Grey solid, mp 237–239 °C (dec), 0.31 g, yield 93%; IR (KBr) (v_max_/cm^−1^): 3305 (NH), 1731 (C=O), 1723 (C=O), 1694 (C=O), 1279 (C–O); ^1^H NMR (250.1 MHz, DMSO-*d*_6_) δ 1.23 (t, ^3^*J* = 7.1 Hz, 3H, CH_3_), 2.24 (s, 3H, CH_3_), 2.41 (s, 3H, CH_3_), 3.81 (s, 3H, OCH_3_), 4.22 (q, ^3^*J* = 7.1 Hz , 2H, OCH_2_), 7.18 (s, 1H, CH), 8.13 (s, 1H, CH), 8.73 (s, 1H, CH), 11.48 (s, 1H, NH) ppm; ^13^C NMR (62.9 MHz, DMSO-*d*_6_) δ 14.3 (CH_3_), 21.1 (CH_3_), 22.4 (CH_3_), 53.5 (OCH_3_), 61.8 (OCH_2_), 115.6 (CH), 118.5(C), 119.3 (C), 120.7 (C), 121.0 (C), 122.0 (C), 122.2 (C), 123.5 (CH), 128.3 (C), 129.4 (CH), 155.2 (C=O), 162.4 (C=O), 165.0 (C=O) ppm; EIMS *m*/*z*: 342 (M^+^, 100), 327 (7), 311 (71), 297 (74), 270 (76); anal. calcd for C_18_H_18_N_2_O_5_ (342.35): C, 63.15; H, 5.30; N, 8.18; found: C, 63.21; H, 5.24; N, 8.11.

#### General procedure for the synthesis of compounds **9a** and **9b**

To a stirred solution of **8** (2 mmol) and **2** (2 mmol) in MeCN (5 mL), was added **3** (2 mmol) at rt. The mixture was heated under reflux for 18 h. The resulting precipitate was filtered off and recrystalized from MeOH to give **9**.

**6-Ethyl 8-methyl 1-oxo-1,2,3,4-tetrahydropyrrolo[1,2-*a*]pyrazine-6,8-dicarboxylate (9a):** Grey solid, mp 220–222 °C (dec), 0.45 g, yield 85%; IR (KBr) (v_max_/cm^−1^): 3276 (NH), 1724 (C=O), 1712 (C=O), 1678 (C=O), 1293 (C–O); ^1^H NMR (250.1 MHz, DMSO-*d*_6_) δ 1.29 (t, ^3^*J* = 7.2 Hz, 3H, CH_3_), 3.54–3.60 (broad, 2H, CH_2_), 3.68 (s, 3H, OCH_3_), 4.01–4.08 (broad, 2H, CH_2_), 4.33 (q, ^3^*J* = 7.2 Hz, 2H, OCH_2_), 7.18 (s, 1H, CH), 7.39 (s, 1H, NH) ppm; ^13^C NMR (62.9 MHz, DMSO-*d*_6_) δ 14.7 (CH_3_), 31.2 (CH_2_), 31.6 (CH_2_), 51.3 (OCH_3_), 61.7 (OCH_2_), 115.5 (CH), 118.1 (C), 120.5 (C), 122.8 (C), 154.3 (C=O), 161.7 (C=O), 165.3 (C=O) ppm; EIMS *m*/*z*: 266 (M^+^, 100), 235 (78), 221 (74), 194 (64); anal. calcd for C_12_H_14_N_2_O_5_ (266.25): C, 54.13; H, 5.30; N, 10.52; found: C, 54.18; H, 5.24; N, 10.48.

**Diethyl 1-oxo-1,2,3,4-tetrahydropyrrolo[1,2-*a*]pyrazine-6,8-dicarboxylate (9b):** Grey solid, mp 218–220 °C (dec), 0.46 g, yield 83%; IR (KBr) (v_max_/cm^−1^): 3282 (NH), 1721 (C=O), 1714 (C=O), 1682 (C=O), 1290 (C–O); ^1^H NMR (250.1 MHz, DMSO-*d*_6_) δ 1.26 (t, ^3^*J* = 7.2 Hz, 3H, CH_3_), 1.35 (t, ^3^*J* = 7.2 Hz, 3H, CH_3_), 3.64 (broad, 2H, CH_2_), 3.89 (q, ^3^*J* = 7.2 Hz, 2H, OCH_2_), 4.08 (broad, 2H, CH_2_), 4.35 (q, ^3^*J* = 7.2 Hz, 2H, OCH_2_), 7.16 (s, 1H, CH), 7.42 (s, 1H, NH) ppm; ^13^C NMR (62.9 MHz, DMSO-*d*_6_) δ 14.2 (CH_3_), 14.6 (CH_3_), 31.5 (CH_2_), 31.7 (CH_2_), 61.3 (OCH_2_), 61.7 (OCH_2_), 115.4 (CH), 117.9 (C), 120.8 (C), 122.6 (C), 154.9 (C=O), 162.3 (C=O), 165.6 (C=O) ppm; EIMS *m*/*z*: 280 (M^+^, 100), 251 (13), 235 (93), 208 (65); anal. calcd for C_13_H_16_N_2_O_5_ (280.28): C, 55.71; H, 5.75; N, 9.99; found: C, 55.79; H, 5.71; N, 9.93.
